# Health Tract, No. 102

**Published:** 1862-09

**Authors:** 


### HEALTH TRACT, No. 102.

### GREED OF GOLD.

When Napoleon, about 1811, desired to build a palace for the King of Rome, near the barrier de Passy, the shop of a poor cobbler, named Simon, stood in the way. Simon having learned what was going on, demanded twenty thousand francs for his tenement. The administrator hesitated a few days, and then decided to give it; but Simon, goaded by the god of gain, now asked forty thousand francs. This sum was more than two hundred times its value, and the demand was scouted. An attempt was made to change the frontage, but being found impossible, they went again to the cobbler, who had raised his price to sixty thousand francs. He was offered fifty thousand, but refused. The Emperor would not give a franc more, and preferred to change his plans. The speculating son of St. Crispin then saw his mistake, and offered his property for fifty thousand francs, forty thousand, thirty thousand, coming down at last to ten thousand. The disasters of 1814 happened, and all thoughts of a palace for the King of Rome were abandoned. Some months after, Simon sold his shop for one hundred and fifty francs, and in a few days after the sale was removed to an insane asylum; disappointed avarice had driven him crazy.

"There was an old man," says an Eastern parable, "who had abundance of gold; the sound of it was pleasant to his ears, and his eye delighted in its brightness. By day he thought of gold, and his dreams were of gold by night. His hands were full of gold, and he rejoiced in the multitude of his chests; but he was faint from hunger, and his trembling limbs shivered beneath his rags. No kind hand ministered to him, nor cheerful voices made music in his home. And there came a child to him, and said: 'Father, I have found a secret. We are rich. You shall not be hungry and miserable any more. Gold will buy all things.' Then the old man was wroth, and said: 'Would you take from me my gold?'"

Many years since, a seafaring man called at a village inn on the coast of Normandy, and asked for supper and a bed. The landlord and landlady were elderly people, and apparently poor. He entered into conversation with them, invited them to partake of his cheer—asked them many questions about themselves and their family, and particularly of a son who had gone to sea when a boy, and whom they had long given over as dead. The landlady showed him to his room, and when she quitted him, he put a purse of gold into her hand, and desired her to take care of it till the morning—pressed her affectionately by the hand, and bade her good night. She returned to her husband, and showed him the gold. For its sake they agreed to murder the traveler in his sleep, which they accomplished, and buried the body. In the morning early, came two or three relations, and asked in a joyful tone for the traveler who had arrived there the night before. The old people seemed greatly confused, but said that he had risen very early and gone away. "Impossible!" said the relations. "It is your own son, who is lately returned to France, and is come to make happy the evening of your days, and he resolved to lodge with you one night as a stranger, that he might see you unknown, and judge of your conduct toward wayfaring mariners." Language would be incompetent to describe the horror of the murderers, when they found that they had dyed their hands in the blood of their long-lost child. They confessed their crime, the body was found, and the wretched murderers expiated their offense by being broken alive upon the wheel.

A London shipping merchant, on a beautiful May morning of 1862, was found dead in his chamber, with so horrible an expression on his countenance, that the persons who first entered the apartment instinctively turned away their faces in uncontrollable terror. Death had given him but a minute's notice, but it was a minute of sane consciousness that he was leaving four millions of dollars; that he would instantly stand before his Maker, to give an account of his stewardship; and that through a long life he had made it his boast and a consistent practice: "I never bestow a penny in charity."

Strive, reader, against the "greed of gold." It is a merciless tyrant, and in the end not only kills the body, but destroys the soul.

